# Media framing and construction of childhood obesity: a content analysis of Swedish newspapers

**DOI:** 10.1002/osp4.150

**Published:** 2018-02-11

**Authors:** J. van Hooft, C. Patterson, M. Löf, C. Alexandrou, S. Hilton, A. Nimegeer

**Affiliations:** ^1^ Department of biosciences and nutrition Karolinska Institutet Huddinge Sweden; ^2^ MRC/CSO Social and Public Health Sciences Unit University of Glasgow Glasgow UK

**Keywords:** Childhood obesity, content analysis, media, Sweden

## Abstract

**Objective:**

Despite lower prevalence than most European countries, childhood obesity is a Swedish public health priority due to its lasting health impacts and socioeconomic patterning. Mass media content influences public and political perceptions of health issues, and media framing of childhood obesity may influence perceptions of its solutions. This study examines framing of childhood obesity in Swedish morning and evening newspapers from 1996 to 2014.

**Methods:**

Content analysis of 726 articles about childhood obesity published in the five most‐circulated Swedish newspapers. Article content coded quantitatively and subjected to statistical analysis, describing relationships between themes and trends over time.

**Results:**

Childhood obesity was consistently problematised, primarily in health terms, and linked to socio‐economic and geographical factors. The yearly frequency of articles peaked in 2004, followed by a decline, corresponding with evidence about prevalence. Childhood obesity was framed as being driven by individual behaviours more frequently than structural or environmental factors. Structural framings increased over time, but constructions of the problem as driven by individual behaviours, particularly parenting, remained prominent.

**Conclusions:**

A relative growth in structural framings of causes and solutions over time, combined with prominent coverage of socio‐economic inequalities, might be indicative of public and political amenability towards societal‐level solutions, but individual behaviours remain prominent in framing of the issue. Health advocates might incorporate these insights into media engagement.

## Introduction

In Sweden, as elsewhere in the developed world, childhood obesity is a public health concern. Approximately 15% of Swedish children are overweight or obese 1, and obesity among 10‐year‐old children in Sweden is four times as prevalent as in the 1980s 2. While prevalence is lower in Sweden than most European countries, 3 and may have stabilised 4, 5, 6, childhood overweight and obesity remains an area of policy concern given its relationships with social deprivation 6, 7 and various negative health outcomes 8.

Globally, obesity is driven by a range of interacting environmental and individual factors, 9 befitting a comprehensive, multilevel package of targeted solutions 10. Despite the necessity of government intervention to address environmental drivers of obesity, critics have argued that governments have predominantly relied upon individuals, the private sector and non‐governmental organisations to solve the problem 9. In trying to enact the ‘hard paternalism’ required to address the obesity problem 10, 11. policymakers can expect resistance from both electorates and corporate interests, while. Conversely the ‘soft paternalism’ of promoting individual responsibility and indirect positive reinforcement of healthy behaviours is less controversial, risky and expensive, but unlikely to sufficiently address the problem 10, 11, 12.

Policy decisions are influenced by perceived public opinion, which is in turn influenced by mass media representations of issues. Media both set the public agenda 13 and construct frames 14, 15 that influence understandings of the definitions of problems; the causes of problems; attribution of blame to, and moral evaluations of, causal agents; the people affected by problems; and the potential solutions to problems 15. Audiences do not absorb media frames uncritically, but media frames influence the construction of individual frames. 15, 16 Therefore, media representations of childhood obesity contribute to public understandings of the obesity issue, including its drivers and solutions, and those understandings may influence public acceptance of legislative interventions. The framing of childhood obesity may differ from that of general obesity because the affected group, children, may be viewed as more vulnerable and less empowered than adults, and therefore potentially constructed as innocent victims, not responsible for their own weight gain 17, 18. While the construction of children as innocent victims intuitively lends itself to a diminished focus on individual‐level solutions to obesity, the issue is complicated by the role of parents; implications of judgement about parental competence 19 and potential conflicts between government advice and lay expertise in parenting decisions 20 may make stakeholders hesitant to apply policy‐level solutions to childhood obesity.

Researchers have studied media representations of obesity, typically focusing on: constructions of the problem; the science of obesity; and obesity policy 21. Analyses of Swedish media content include Sandberg's 22 examination of Swedish daily newspapers' framing of obesity, and Roos' 23 comparison of Swedish and UK newspaper representations of obesity prevention. Neither study focused on obesity in children. Sandberg 22 analysed 1,925 articles published between 1997 and 2001, highlighting the construction of two conflicting frames, with obesity characterised as either a health problem or a cosmetic concern. While Sandberg's 22 analysis does not focus on constructions of drivers and solutions, she identifies a lack of advice about solving the problem, and finds that the food industry are neither engaged in, or held to account by, news coverage. Roos 23 analysed a relatively small sample of 199 articles about obesity from the UK's *The Guardian* newspaper and 24 from Sweden's *Dagens Nyheter*, identifying the stakeholders associated with obesity and its prevention, and the prevention measures described. Roos 23 identified greater acceptance of the government's role in preventing obesity in the Swedish publication than the UK publication, attributing this to a reflection of a Nordic welfare state.

Literature on media representations of childhood obesity covers a broad range of focuses, including how media framing has defined obesity in terms of its causes and potential solutions 24, and the gendered nature of parental blame 19. Boero's 21 review of social science perspectives on obesity media suggests that individual framings tend to dominate representations of obesity, but identifies childhood obesity as a possible exception, with blame for children's overweight shifted to parents and societal institutions. Recent work in Ireland by De Brún emphasises the gendered nature of this parental blame, with mothers bearing the brunt of responsibility 19.

This study is intended to enhance understandings of media representations of obesity, with a specific focus on childhood obesity in Sweden. The research aims are to analyse the frequency of Swedish newspapers' coverage of childhood obesity and examine how obesity is framed in terms of defining the problem, attributing causes and presenting solutions.

## Methods

The methods were largely based on an earlier study of UK newspaper coverage of obesity 18.

### Publication selection

To ensure articles were taken from a broad selection of Swedish newspapers with high circulations 25, three morning publications were selected (*Dagens Nyheter*, *Svenska Dagbladet* and *Göteborgs‐Posten*), in addition to two evening publications, including their Sunday editions (*Aftonbladet*, *Aftonbladet Söndag*, *Expressen*, and *Expressen Söndag*). Morning newspapers are read by 63% of the population aged between 15 and 79 years old (predominantly people aged 65–79 with high incomes), while evening newspapers are read by 27% of the population aged 15–79 (predominantly middle‐income readers aged 25–44) 25.

### Article search and manual exclusion

Articles were retrieved from the *Retriever Research* (*Mediearkivet*) database. A search period beginning 1 January 1996 and ending 31 December 2014 was chosen to match the starting point of Hilton and colleagues' 18 study and include a further two years of more recent coverage. The search string, which was designed to identify articles containing terms related to both children and obesity, was “(barn) AND (“tjock” OR “övervikt” OR “fetma”), which translates to “(child) AND (“fat” OR “obese” OR “obesity”). The initial search retrieved 3,919 articles. Inclusion and exclusion criteria were applied manually to each article. To be included, articles were required to: be at least 50% focused on human obesity; mention obesity in children aged 0–18; and be printed in the news, comment, feature, business, city, sport, travel or home sections of their publications. Articles were excluded if they: were printed in readers' letters or television guide sections; were front page lead paragraphs whose content overlapped with longer articles in the body of the paper; or primarily focused on dieting for cosmetic reasons. The final sample comprised 726 articles.

### Coding

The relevant manifest content 26 of each article was coded manually using a coding frame initially developed by Hilton and colleagues 18, adapted for Swedish news content. Basic information recorded included: publication date; headline; page number; source publication; and terms used to describe society's obesity problem. Broad thematic categories recorded included definitions of the obesity problem, drivers of the obesity problem, and potential solutions to the obesity problem (see Table 2). These categories partially followed Entman's conceptualisation of framing 15, with the exception of the aspect of moral evaluations of parties responsible for the problem.

Additionally, each article's headline was coded as either alarmist, reassuring or neutral in tone, based on the coder's interpretation of the author's intent. As such, headlines were not coded as ‘alarmist’ or ‘reassuring’ simply for communicating ‘bad’ or ‘good’ news, rather they were coded for the presence of language that the coders interpreted as having been selected with the intention of provoking either alarm or reassurance. For example, the headline *‘1 in 4 children obese’* would be coded as neutral, while *‘Obesity catastrophe as 1 in 4 children obese’* would be coded as alarmist.

All articles were coded by JvH. Where ambiguities arose during coding, articles were translated into English and additionally coded by AN and SH, and discussed to reach shared understandings of the definitions of each code, which were recorded in a coding guide. Following initial coding, a random sample of 10% (*n* = 70) of articles were independently double‐coded by CA using the coding guide for reference.

### Analysis

Data were entered into STATA for statistical analysis. Cohen's kappa tests were used to test agreement between JvH and CA in coding each variable. Kappa coefficients for each variable are listed in Table 2. Three variables with kappa coefficients below 0.6 (‘substantial’ agreement 38) were excluded: “Swedish healthcare service is failing obese”; “Obesity is an economic cost to society”; and “Identified poor food labelling, education”.

Descriptive statistics are presented in frequency tables. Statistical procedures comprised chi‐squared tests (to examine relationships between publication category and mentions of specific themes and terms) and simple logistic regressions (to examine trends in reporting on different categories of drivers and solutions over time). The threshold for statistical significance was set at *p* < 0.05 throughout, and 95% confidence intervals are reported where appropriate.

## Results

### Article characteristics

Table 1 summarises the frequency of articles and front‐page articles by publication, and illustrates the variation in word length of the articles. In total, 726 articles were coded, 61.7% published in morning newspapers and 38.3% in evening newspapers. Only five articles (0.7%) were published on front pages, and only in morning newspapers, although, as noted in the methods, that figure excludes brief, front‐page lead‐ins highlighting full‐length articles from other pages, such that front‐page prominence of the issue of childhood obesity may have been greater than our data suggest. The shortest article was 23 words long, and the longest 2,704. The median word count of all articles was 630.

**Table 1:** – Summary of article characteristics

**Newspaper category**	**Newspaper title**	**Total articles**		**Front page articles** *		**Word count**		
		**n**	**% (95% CI)**	**n**	**% (95% CI)**	**Lower quartile**	**Median (50%)**	**Upper quartile**
Morning	Svenska Dagbladet	134	18.5 (15.6–21.3)	1	0.7 (0.0–2.2)	225	375	603
	Dagens Nyheter	132	18.2 (15.4–21.0)	0	0	356	494	817
	Göteborgs‐Posten	182	25.1 (21.9–28.2)	4	2.2 (0.0–4.3)	236	386	533
Evening	Aftonbladet & Aftonbladet Söndag	170	23.4 (20.3–26.5)	0	0	193	380	690
	Expressen & Expressen Söndag	108	14.9 (12.3–17.5)	0	0	300	474	638
	Total	726	100	5	0.7 (0.1–1.3)	246	417	630

* Front‐page articles were only included in the sample if part or all of the main content was featured on front pages; brief front‐page lead‐ins to full‐length articles published elsewhere in issues were not included.

**Figure 1:**
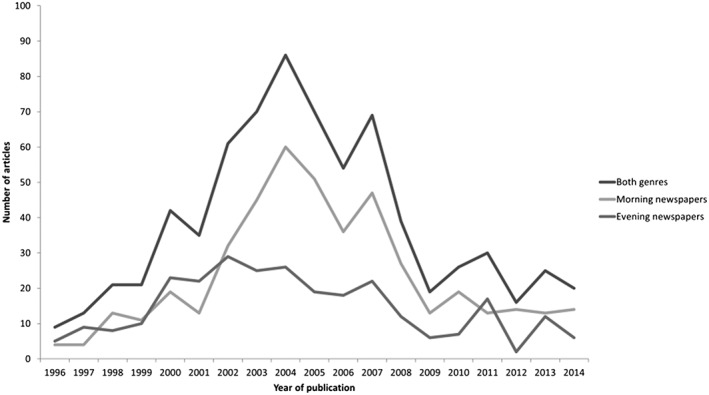
Number of articles reporting on childhood obesity published by category per year

Figure 1 illustrates the frequency of articles reporting on childhood obesity within the sample publications by publication year. The quantity of reporting grew between 1996 and 2002, and the increase was more pronounced in morning newspapers. The frequency of articles was greater at the end of the sample period in 2014 (*n* = 20) than the beginning of the period in 1996 (*n* = 9), but the frequency of reporting across the sample period was characterised by an inverse U‐curve; coverage grew to a peak of 86 articles in 2004, before falling sharply, and then levelling‐off from 2009 to 2014. The period of growth from 2000 to 2004 coincided with various events that received heightened media coverage, including: a WHO report on the obesity epidemic 27 in 2000; evidence of a long‐term rise in childhood overweight in children 2, 28; and the documentary film *Supersize Me* in 2004.

### Headline tone

The majority of headlines were rated “neutral” in tone (*n* = 637, 92.7%), while 40 (5.51%) were rated “alarmist” and eight (1.1%) “reassuring”. Five (0.7%) articles did not have headlines, but were retained in the sample because they had their own records in the *Retriever Research* database and appeared to be complete articles. Morning newspaper headlines were rated as neutral (*n* = 45, 94.9%) significantly (*p* = 0.004) more frequently than evening headlines (*n* = 248, 89.2%), while evening headlines were significantly (*p* < 0.001) more alarmist (*n* = 27, 9.7%) than morning headlines (*n* = 13, 2.9%).

### Definitions of the childhood obesity problem

**Figure 2:**
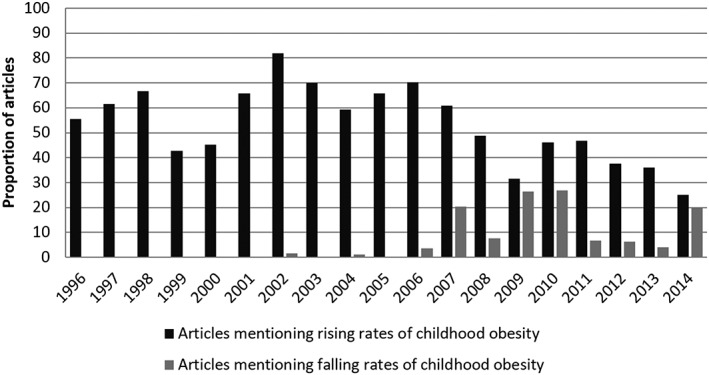
Changing proportions of articles presenting childhood obesity rates as rising or declining

Table 2 itemises the frequency of articles mentioning thematic categories, including different definitions of obesity. More articles (47.7%) mentioned obesity rates outside Sweden than those that quantified rates within Sweden (36.6%) (Table 2). More than half of the articles mentioned a past, present or predicted future rise in childhood obesity prevalence (*n* = 425, 58.5%), while 41 (6.5%) mentioned declining or plateauing prevalence, largely subsequent to 2007. Figure 2 displays the proportion of articles reporting on rising or declining prevalence, illustrating a relative increase in mentions of a decline towards the end of the sample period.

**Table 2:** ‐ Frequency of articles mentioning specific definitions of, drivers of and solutions to childhood obesity

Thematic code	n	% (95% CI)	Inter‐rater agreement†
**Problem definitions**			
Rates in Sweden	266	36.6 (33.1–40.2)	0.9
Rates outside Sweden	346	47.7 (44.0–51.3)	0.9
Increase/rise in rates	425	58.5 (54.9–62.1)	0.9
Decrease/drop in rates	41	5.6 (3.9–7.3)	1.0
Obesity as a risk to health	349	48.1 (44.4–51.7)	0.9
Obesity as a cosmetic problem	47	6.5 (4.7–8.3)	0.7
Obesity burden's Swedish healthcare system	54	7.4 (5.5–9.4)	0.6
Socio‐economic and geographic differences	210	28.9 (25.6–32.2)	0.9
Problem lies with women, teenage girls	117	16.1 (13.4–18.8)	0.8
Problem lies with men, teenage boys	118	16.3 (13.6–18.9)	0.8
Obesity is not a problem	18	2.5 (1.3–3.6)	1.0
Discrimination, bullying, stigmatisation	139	19.1 (16.2–22.0)	0.9
**Drivers of obesity**			
*Overall drivers*			
Any drivers mentioned	632	87.1 (84.6–89.5)	‐
Any biological/genetic driver mentioned	125	17.2 (14.5–20.0)	0.8
Any individual driver mentioned	522	71.9 (68.6–75.2)	‐
Any societal driver mentioned	466	64.2 (60.7–67.7)	‐
*Individual drivers*			
Mentions poor diet, overeating	217	29.9 (26.6–33.2)	0.6
Mentions dieting as a driver of obesity	55	7.6 (5.6–9.5)	0.6
Self‐control, willpower and choices	239	32.9 (29.5–36.3)	0.6
Lack of exercise, sedentary lifestyle	290	39.9 (36.4–43.5)	0.8
Identifies a lack of parenting	216	29.8 (26.4–33.1)	0.8
*Societal drivers*			
Abundance of processed/fast food	345	47.5 (43.9–51.2)	0.8
Lack of health services, facilities	165	22.7 (19.7–25.8)	0.8
Food/drink advertising and promotions	110	15.2 (12.5–17.8)	1.0
Identifies normalisation of obesity	43	5.9 (4.2–7.6)	0.9
Technological changes, modern living	77	10.6 (8.4–12.9)	0.7
**Solutions to obesity**			
Any solution mentioned	589	81.1 (78.3–84.0)	‐
Biological	84	11.6 (9.2–13.9)	1.0
Individual	407	56.1 (52.4–59.7)	0.8
Societal	387	53.3 (49.7–56.9)	0.8

† Cohen's kappa test of inter‐rater agreement. Agreement was not calculated for variables that were derived from other variables

In characterising the specific nature of the obesity problem, almost half of articles identified obesity as a risk to health (*n* = 349, 48.1%). Other definitions included obesity as a burden to the healthcare system (*n* = 54, 7.4%) or a cosmetic problem (*n* = 47, 6.5%). A small minority of articles (*n* = 18 2.5%) characterised obesity as not a problem. This is in keeping with seminal research on news values by Galtung and Ruge (1965) that suggests that negative stories are more likely to be considered newsworthy by reporters than positive stories 29. Almost one‐third of articles (*n* = 210, 28.9%) associated obesity with socioeconomic and geographical inequalities, with morning articles doing so significantly more frequently (*p* = 0.001). Discrimination, bullying and stigma were reported as aspects of the obesity problem in 139 (19.1%) articles, and significantly more frequently in evening articles (*p* = 0.002). Comparable proportions of articles mentioned men/boys (*n* = 118, 16.3%) and women/girls (*n* = 117, 16.1%) in relation to the obesity problems. Of the 216 (29.8%) articles that mentioned parenting as a driver of obesity, significantly (*p* < 0.001) more mentioned men or boys (*n* = 42, 19.4%) than women or girls (*n* = 30, 13.9%).

### Drivers

Most (*n* = 632, 87.1%) articles mentioned at least one potential driver of obesity (Table 2). The most commonly mentioned category of driver was individual drivers (*n* = 522, 71.9%), followed by societal (*n* = 466, 64.2%) and biological drivers (*n* = 125, 17.2%). A breakdown of specific drivers can be found in Table 2.

### Solutions

Most articles mentioned one or more potential solution to the obesity problem (*n* = 589, 81.1%) (Table 2). More than half mentioned individual solutions (e.g. diet, physical activity or parenting) (*n* = 407, 56.1%), while a similar proportion mentioned societal solutions (e.g. weight loss interventions, school initiatives) (*n* = 387, 53.3%), and 84 (11.6%) mentioned biological solutions (e.g. pharmaceutical or surgical interventions). While individual solutions were mentioned more frequently than societal solutions, the 95% confidence intervals of the two variables overlapped (Table 2). Only societal solutions differed significantly by newspaper category, being mentioned more often by morning newspapers than evening newspapers (*p* < 0.001).

### Trends in representations of drivers and solutions

**Figure 3:**
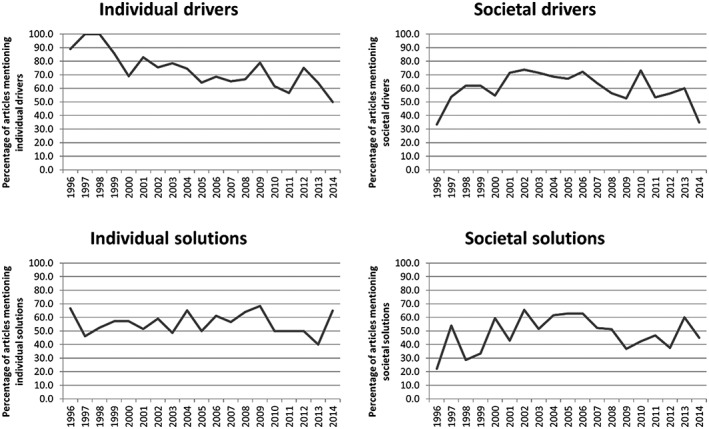
Proportion of articles mentioning individual drivers, societal drivers, individual solutions and societal solutions by publication year

Trends in the proportion of articles mentioning different categories of drivers and solutions over time were analysed (Figure 3). Across the sample period of 1996 to 2014, publication year was only a statistically significant predictor of articles mentioning individual drivers, mentions of which decreased over the 19‐year period (regression coefficient − 0.093, p < 0.001). Mentions of individual solutions remained relatively stable (coefficient 0.004, *p* = 0.827). Mentions of societal drivers exhibited an inverse U‐curve, increasing from 1996 to 2002 (coefficient 0.206, *p* = 0.012) before declining from 2002 to 2014 (coefficient − 0.087, *p* = 0.001). Similarly, mentions of societal solutions increased from 1996 to 2002 (coefficient 0.241, *p* = 0.004) before declining towards the end of the sample period (coefficient − 0.062, *p* = 0.013), punctuated by smaller peaks of 63.0% in 2006 and 60.0% in 2013.

## Discussion

This analysis of 726 Swedish newspaper articles about childhood obesity produced key findings related to the changing frequency of coverage; the newsworthiness of the topic; definitions of the problem; and representations of drivers and solutions. The frequency of articles rose steadily from 1996 to 2004, and subsequently declined until 2014. From the perspective of agenda‐setting theory, the rise in prominence of the issue of childhood obesity within mass media likely led to an increase in the salience of that issue in public audiences' consciousness, while the decline in prominence likely precipitated a decline in public attention 13. Childhood obesity was found to be a relatively low‐profile issue, evidenced by a low proportion of front‐page articles. Articles predominantly presented childhood obesity as a growing health problem. Finally, while individual framing was more frequent than societal framing, there was evidence of a long‐term shift decrease in framing childhood obesity as individually‐driven. Evening and morning newspapers varied significantly in certain aspects of content, suggesting that different segments of the Swedish population may receive different messages about childhood obesity. While the older, higher‐income readers of morning newspapers 25 were more likely to read about childhood obesity's socioeconomic and geographical patterning, as well as its societal solutions to obesity, the younger, lower‐income readers of evening newspapers 25 where more likely to encounter alarmist headlines.

While the prevalence of childhood obesity is predicted to continue rising internationally 8, recent evidence suggests that prevalence in Sweden may have plateaued since approximately 2003 4, 6. This is consistent with the contemporaneous decline in newspaper coverage of the issue. Similarly, while most articles, particularly in morning publications, presented childhood obesity as increasing, not decreasing, more articles reported declining prevalence towards the end of the sample period. 5, 30 These trends may result from media attention following evidence (and granting more salience to ‘bad’ news than ‘good’), but the data cannot provide causal evidence of this.

While research has demonstrated that US 31 and UK 18 reporting on obesity grew to similar peaks in 2003 and 2004, neither study extended as far as 2014, and neither focused on childhood obesity specifically, so it is yet to be determined whether Swedish publications' decline in coverage in also occurred elsewhere. However, in Hilton and colleagues' 18 study of general obesity in UK newspapers, the initial peak in coverage (of general obesity) in 2004 was not followed by a steady decline, but by higher peaks in 2006–2008. The UK authors attributed the initial peak to reaction to a 2003 WHO report on causes of, and solutions to, the obesity problem 32, which may have also influenced Swedish discourse.

The research design does not allow direct comparison of Swedish newspapers' coverage of childhood obesity with that of other issues, but the finding that less than 1% of articles were published on front pages suggests that childhood obesity was not a high‐profile issue. By comparison, research suggests that coverage of obesity 18 and various other health topics 33 in other countries included higher proportions of front‐page news, and one study of general health content coverage in Swedish newspapers suggested that around 10% of general health content was granted front page salience 34. Sandberg 22, who also found that a small proportion of obesity stories in Swedish newspapers were published on front pages, remarks that this low media profile is incongruent with the extent of the impacts of obesity. When comparing the proportion of front‐page articles, it must be noted that the sample analysed here did not include brief front‐page lead‐ins to full‐length articles published elsewhere within issues. As such, some high‐profile ‘teasers’ related to childhood obesity may have been featured on front pages without being reflected in the data.

Obesity was characterised as a risk to health in almost half of the articles. That more than half of articles did not overtly identify health as a risk could be interpreted as supporting Sandberg's 22 conclusion that Swedish newspapers are failing readers through poor risk communication. Sandberg 22 specifically identified an imbalance between framing obesity as either a health problem or a beauty problem. Just 6.5% of articles in the present study mentioned obesity as a cosmetic problem, compared to 39% in Sandberg's 22, but this difference may be an artefact of excluding articles focused on dieting for cosmetic reasons.

Analysis found negligible difference in the proportions of mentions of males and females within articles, suggesting that coverage was relatively gender‐neutral, which may be particularly notable given the frequent mention of parenting. Zivkovic and colleagues 35 identified gender inequalities within Australian media coverage of childhood obesity, albeit related to parents rather than children, identifying childhood obesity as a point at which societal discourses about gender, parenting, children and the state intersect. However, Zivkovic and colleagues' 35 findings cannot be directly compared with the present study, as the coding in this study did not differentiate mothers from females more generally. An alternative interpretation of the frequency of mentions of males and females within Swedish newspaper coverage of childhood obesity may be that it is symptomatic of a tendency for coverage to eschew personalised accounts of childhood obesity in favour of more general, impersonal coverage. Such impersonal coverage could be interpreted as an contributing to the othering and dehumanisation of those classified as obese, as has been identified in other analyses of media representations of obesity 36. Conversely, a tendency towards impersonal representations of childhood obesity, in comparison to adult obesity, may be symptomatic of a desire to avoid stigmatising young people due to perceptions of their ‘innocence’. For a more complete understanding of these issues it would be necessary to analyse media content with greater focus on the aspect of gender, potentially taking a qualitative approach.

The relative prominence of individual‐level drivers over societal‐level drivers is consistent with evidence from other countries 18, 21, 24, 31. Examining trends in reporting of drivers and solutions revealed a significant decline in reporting on individual drivers, while individual solutions, societal drivers and societal solutions remained relatively stable across the sample period. The decrease in reporting on individual drivers across the sample period echoes that found in UK 18 and US 31 newspapers, suggesting that a shift from individual to societal framings of obesity is not unique to one country. Nonetheless, despite evidence of change, individual drivers remained more frequently‐mentioned than societal drivers by the end of the sample period, suggesting that optimism about a more favourable policy climate should be cautious. News media's predisposition to individual framings may be deep‐rooted, due to, as Bastian 24 suggests, a desire to cater to readers that are perceived to want to read about concrete situations in which they can identify themselves, rather than the more ‘abstract and elusive’ (p.138) societal aspects of obesity.

Bastian's 24 analysis of both academic literature and Australian newspaper coverage of childhood obesity identified an emphasis on societal framings in the former, and individual framings in the latter. Lawrence's 37 analysis of framing of obesity in US news articles and other texts found that, in the two decades preceding 2004, obesity frames shifted from individualised and medicalised conceptions to a greater focus on environmental causes. Lawrence considers whether this reframing is likely to have created a climate more favourable to policy solutions, and concludes that, while there was a more prominent focus on environmental causes, obesity continued to be defined as a risk to specific societal groups, rather than a risk to all, which may obstruct acceptance of population‐level legislative solutions 37. Hilton and colleagues 18 analysed content of 2,414 UK newspaper articles about obesity, identifying a rise in coverage of obesity between 1996 to 2010, particularly in articles mentioning children. Reinforcing Lawrence's 37 US‐based conclusions with quantitative data, they identified trends away from a focus on individual solutions towards greater prominence of societal solutions, which they conclude might be indicative of growing national public familiarity with addressing the obesogenic environment through legislation 18.

This study has several limitations. The analysis focuses exclusively on print newspaper articles excluding broadcast news, online news and social media. As such, the data did not represent a comprehensive cross‐section of public discourse around childhood obesity. Kim and Willis 31 found that television news had a greater focus on personal responsibility than did newspapers, illustrating that there can be important differences in framing between media. However, newspapers remain a central part of the Swedish news media landscape 25. A second limitation emerged from comparison with Sandberg's 22 findings; the focus on collecting frequencies of occurrences of manifest content within articles facilitates a statistical overview of relative frequencies and trends within a large sample, but does so at the cost of deeper, qualitative interpretation of the latent content of the text. The understandings provided by this study could be expanded upon through qualitative analysis of the themes analysed at a higher level within this study. Finally, while a randomly‐selected 10% of the sample was double‐coded, and inter‐rater agreement calculated to identify and remove inconsistently‐coded variables, double‐coding the whole sample may have mitigated the risk of coder bias more effectively.

Taking limitations into account, this study represents a thorough, manual content analysis of a relatively large sample of articles from a broad selection of highly‐circulated publications, documenting Swedish newspaper frames of childhood obesity. It is important to understand contexts like Sweden's, in which childhood obesity prevalence is relatively low, as a benchmark for international comparison. One useful focus of future research could be to analyse trends in individual and societal framing of childhood obesity across several countries, to further investigate the extent to which the shift towards societal framing identified in this study is an international phenomenon. This study found evidence of a shift in media discourse around childhood obesity from individual to societal framings, which, literature suggests, could lead to a policy environment more amenable to enacting the legislative solutions necessary to address obesity 10, 11, 37. However, despite that trend, a focus on individual‐level drivers, particularly parental behaviours, remains dominant in Swedish newspapers' framing of childhood obesity.

## Funding

CP, AN and SH's time for this research was funded by the Informing Healthy Public Policy programme (Funded by the Medical Research Council MC_UU_12017–15 and the Chief Scientist Office SPHSU15) of the MRC/CSO Social and Public Health Sciences Unit, University of Glasgow. The funding bodies had no role in the design, collection, analysis or interpretation of this study.

## Disclosure

Chris Patterson, Amy Nimegeer and Shona Hilton's time was funded by core funding from the Medical Research Council (MC_UU_12017–15) and the Chief Scientist Office (SPHSU15) as part of funding for 5‐year programme of research on ‘Informing Healthy Public Policy’ as part of MRC/CSO SPHSU Quinquennial Core Funds. During this time Shona Hilton also declares grants related to health and the media from the National Institute of Health Research and the Cancer Research UK. Jutta van Hooft, Marie Löf and Christina Alexandrou declare no conflicts of interest.
